# High fibroblast-activation-protein expression in castration-resistant prostate cancer supports the use of FAPI-molecular theranostics

**DOI:** 10.1007/s00259-021-05423-y

**Published:** 2021-07-05

**Authors:** Claudia Kesch, Leubet Yirga, Katharina Dendl, Analena Handke, Christopher Darr, Ulrich Krafft, Jan Philipp Radtke, Stephan Tschirdewahn, Tibor Szarvas, Ladan Fazli, Martin Gleave, Frederik L. Giesel, Uwe Haberkorn, Boris Hadaschik

**Affiliations:** 1 Department of Urology, University of Duisburg-Essen, and German Cancer Consortium (DKTK), University Hospital Essen, Hufelandstrasse 55, 45147 Essen, Germany; 2 Department of Nuclear Medicine, University Hospital Heidelberg, Heidelberg, Germany; 3 Department of Urology, Semmelweis University, Budapest, Hungary; 4 Vancouver Prostate Center, Vancouver General Hospital and Department of Urologic Sciences, University of British Columbia, Vancouver, Canada; 5 Clinical Cooperation Unit Nuclear Medicine, German Cancer Research Center (DKFZ), Heidelberg, Germany; 6 Department of Nuclear Medicine, University Hospital Düsseldorf, Düsseldorf, Germany

**Keywords:** Fibroblast-activation-protein, Castration-resistant prostate cancer, [^68^ Ga]Ga-FAPI-04 PET/CT, Prostate cancer

## Abstract

**Purpose:**

To evaluate fibroblast-activation-protein (FAP) expression in different clinical stages of prostate cancer (PC) with regards to utility of [^68^ Ga]Ga-FAPI-04 PET/CT imaging in patients with castration-resistant PC (CRPC).

**Methods:**

Tissue microarrays (TMAs) were constructed from prostatic tissue from 94 patients at different stages of PC (primary PC, patients undergoing neoadjuvant androgen deprivation therapy, CRPC, and neuroendocrine PC (NEPC)) and were stained with anti-FAP monoclonal antibody. A positive pixel count algorithm (H-Index) was used to compare FAP expression between the groups. Additionally, three men with advanced CRPC or NEPC underwent [^68^ Ga]Ga-FAPI-04 PET/CT, and PET positivity was analyzed.

**Results:**

The mean H-index for benign tissue, primary PC, neoadjuvant androgen deprivation therapy before radical prostatectomy, CRPC, and NEPC was 0.018, 0.031, 0.042, 0.076, and 0.051, respectively, indicating a significant rise in FAP expression with advancement of disease. Corroborating these findings [^68^ Ga]Ga-FAPI-04 PET/CT was highly positive in men with advanced CRPC.

**Conclusion:**

Increased FAP tissue expression supports the use of FAP inhibitor (FAPI)-molecular theranostics in CRPC.

**Supplementary Information:**

The online version contains supplementary material available at 10.1007/s00259-021-05423-y.

## Introduction

Besides cancer cells, malignant lesions consist of a tumor microenvironment (TME), the so-called stroma comprising a variety of heterogeneous cell types like immune cells, endothelial cells, fibroblasts, and their extracellular products. It is increasingly becoming apparent that the TME holds an important role in tumorigenesis, tumor neo-angiogenesis, and cancer progression [1]. Cancer-associated fibroblasts are the primary stromal cells within the TME [2] and can be identified based on the expression of various ‘CAF markers’ such as fibroblast-activation-protein (FAP), platelet-derived growth factor receptor ß (PDGFRß), and alpha smooth muscle actin (αSMA), which separates them from the large pool of quiescent fibroblasts present in the body [3]. FAP is a 97-kDa type II transmembrane serine protease [4], and its expression in normal tissue is usually low or undetectable. However, it is overexpressed in many cancers, including 90% of epithelial carcinomas [5–7]. Thus, it is hardly surprising that FAP is increasingly explored as pan-cancer imaging and therapeutic target. Most recently, a family of quinoline-based positron emission tomography (PET)/computed tomography (CT) tracers were derived from a FAP inhibitor (FAPI) and demonstrated promising uptake in multiple cancer entities, including prostate cancer (PC) [8–10]. Nevertheless, until now, little is known about FAP expression in PC and its various stages of disease.

The aim of this study was to evaluate FAP expression in different clinical stages of PC. Our hypothesis that [^68^ Ga]Ga-FAPI-04 PET/CT might be especially useful in castration-resistant PC (CRPC) is further corroborated by clinical case examples.

## Material and methods

### Patients

All patients gave written informed consent to data analysis, bio-banking, and tissue evaluation. Data analysis and biobank reposition were approved by the University of British Columbia, Office of Research Ethics, Clinical Research Ethics Board (UBC CRBE number H09-01,628).

The patients undergoing [^68^ Ga]Ga-FAPI-04 PET/CT gave written informed consent for the procedure following the regulations of the German Pharmaceuticals Act §13(2b). Retrospective data analysis was approved by the Ethics Committee of the University Hospital Heidelberg (S016/2018).

### Tissue microarray construction and immunohistochemistry

Tissue microarrays (TMAs) were constructed from paraffin blocks of prostatic tissue from patients at different stages of PC (primary PC, patients after undergoing neoadjuvant androgen deprivation therapy, CRPC, and neuroendocrine PC (NEPC)) using a manual arrayer (Beecher Instruments, Inc., Silver Springs, MD, USA) with tissue core diameters of 0.6 mm per case [11, 12]. Cores were taken from primary radical prostatectomy (RP) specimens, salvage RP specimens, or palliative TUR-P tissue. H&E-stained slides were reviewed for each case. Areas containing tumor tissue were marked on both the slides and corresponding paraffin blocks for TMA construction. A total of 34 cores of benign prostatic tissue taken from RP specimens in patients undergoing RP for primary PC were also included in the TMA. Reassessment of histopathology in a contiguous H&E-stained TMA section assured the presence of PC/benign tissue and the fidelity of the intended TMA core. Immunohistochemical staining with anti-FAP monoclonal antibody (Abcam, Cambridge, UK) was performed at a concentration of 1:100. Neat DISCOVERY Anti-Rabbit HQ (Roche, Basel, Swiss) was used as secondary antibody and neat DISCOVERY Anti-HQ HRP for detection. All stained slides were digitalized with the SL801 autoloader and Leica SCN400 scanning system (Leica Microsystems) and were subsequently stored in the SlidePath digital imaging hub (DIH; Leica Microsystems) of the Vancouver Prostate Centre. Using the Aperio Image Analysis immunohistochemistry (IHC) (Leica Biosystems), a dedicated uropathologist (LF) selected areas of interest, defined the parameter, optimized the level of intensity, and selected the Positive Pixel Count algorithm for the biomarker (H-Index).

### Radiopharmaceuticals and PET/CT imaging and evaluation

Synthesis and labeling of [^68^ Ga]Ga-FAPI-04 have been previously described [13]. Following the regulations of the German Pharmaceuticals Act §13(2b), the indication for the exam and labeling of the FAPI tracers was done under the direct responsibility of the applying physician. PET/CT imaging and evaluation has been described previously [9, 10] and is specified in the supplements.

### Statistical analysis

Statistical analysis was performed using the GaphPad Prism 8 software. Differences between groups were compared by one-way ANOVA followed by Tukey’s multiple comparison test. The threshold for statistical significance was set at *p ≤ 0.05 and ** p ≤ 0.01. Data represent mean values ± SEM.

## Results

A total of 185 cores from 94 tissue samples of patients undergoing treatment for PC at Vancouver General Hospital were used to build the TMAs. On an average, 2 (range: 1–4) cores per case were assessed. Mean H-index per case was used for further analysis in case of identical core histopathology. Patients undergoing neoadjuvant therapy received a median of 8 month (range: 2–24) androgen deprivation therapy before RP. Patients with CRPC experienced PSA relapse with castrate testosterone levels.

**Fig. 1:**
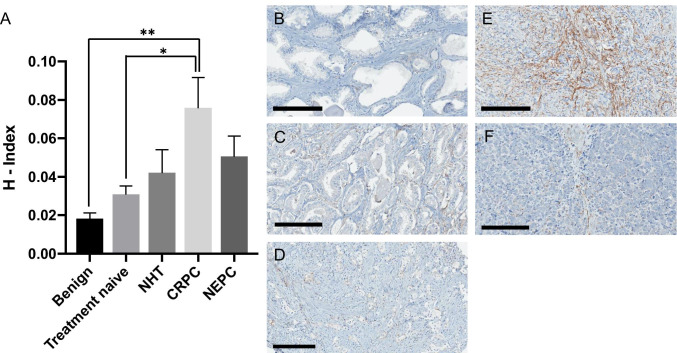
FAP stromal tissue expression. (**A**) Mean fibroblast-activation-protein (FAP) stromal tissue expression levels (H-index) in benign samples (benign) (n = 34), samples from patients with primary prostate cancer (treatment-naive) (n = 36), neoadjuvant androgen deprivation therapy before radical prostatectomy (NHT) (n = 27), castration-resistant prostate cancer (CRPC) (n = 44) and neuroendocrine prostate cancer (NEPC) (n = 44); corresponding, representative images of IHC staining against FAP, (**B**) benign, (**C**) treatment-naive, (**D**) NHT, (**E**) CRPC, and (**F**) NEPC; scale bar 200 μm

The mean H-index for benign tissue (n cores = 29), primary PC (n cores = 36), neoadjuvant androgen deprivation therapy before RP (n cores = 27), CRPC (n cores = 44), and NEPC (n cores = 44) was 0.018 (95% CI 0.012–0.024), 0.031 (95% CI 0.022–0.040), 0.042 (95% CI 0.017–0.068), 0.076 (95% CI 0.043–0.109), and 0.051 (95% CI 0.029–0.073), respectively, indicating a significant rise in FAP expression with advancement of disease. Especially, CRPC samples demonstrated a higher FAP expression compared to benign (p = 0.002) and treatment-naive samples (p = 0.028) (Fig. 1).

**Fig. 2:**
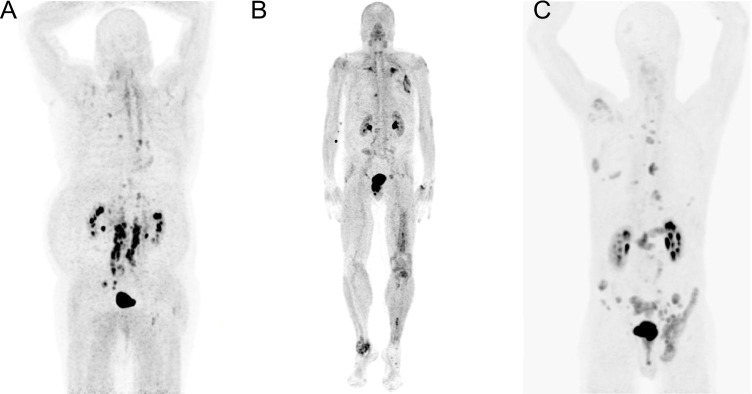
[^68^ Ga]Ga-FAPI-04 PET/CT in patients with castration-resistant prostate cancer. Maximum intensity projections of three patients undergoing [^68^ Ga]Ga-FAPI-04 PET/CT: (**A**) A 77-year-old patient diagnosed with PC in 2001, progressing after standard androgen deprivation therapy, abiraterone, docetaxel, enzalutamide, cabazitaxel, and [^177^Lu]Lu-PSMA-617-RLT; [^68^ Ga]Ga-FAPI-04 PET/CT demonstrating bone and LN metastases; another image of this patient has been published previously [14]; (**B**) A 70-year-old patient diagnosed with PC in 2007 undergoing radical prostatectomy (pT3b, pN1, R1, GS 5 + 3 = 9, ISUP 5, M0) followed by radiation to the prostatic bed and pelvic LN, first-generation antiandrogen therapy, multiple resections of pulmonary metastases, docetaxel chemotherapy, and enzalutamide, finally progressing under treatment with olaparib (confirmed somatic BRCA2 mutation); [^68^ Ga]Ga-FAPI-04 PET/CT demonstrating bone and pulmonary metastases; (**C**) A 71-year-old patient initially diagnosed with metastatic PC in 2016 presenting a mixed phenotype of 20% adenocarcinoma and 80% neuroendocrine cancer. Currently progressing after treatment with three-cycle cisplatin/etoposide chemotherapy and immunotherapy (nivolumab + ipilimumab); [^68^ Ga]Ga-FAPI-04 PET/CT demonstrating bone and LN metastases; a different image of this patient has been published previously in another context [10]

In our clinical pilot study, two of the patients that underwent ^68^ Ga-FAPI PET/CT were progressing after standard treatment for CRPC. ^68^ Ga-FAPI PET/CT demonstrated multiple metastatic lesions confirmed by conventional morphological imaging (CT). One patient (Fig. 2A) (injected activity 232 MBq) demonstrated parailiacal, paraaortal, and mediastinal lymph nodes (LN) metastases (mean maximal standard uptake value SUVmax = 12.58 and SUVmean = 7.38) and bone metastases (SUVmax = 8.45 and SUVmean = 5.04). The other one (Fig. 2B) (injected activity 217 MBq) demonstrated lung metastases (SUVmax = 6.30 and SUVmean = 3.78) and bone metastases (SUVmax = 5.90 and SUVmean = 3.38). The third patient was diagnosed with a mixed adenocarcinoma/neuroendocrine cancer phenotype (Fig. 2C) (injected activity 249 MBq) progressing after chemotherapy and immunotherapy. ^68^ Ga-FAPI PET/CT demonstrated LN (SUVmax = 7.19 and SUVmean = 4.19) and bone metastases (SUVmax = 10.09 and SUVmean = 5.91).

## Discussion

Targeting components of the TME like FAP is an emerging pan-cancer diagnostic and therapeutic strategy. A meta-analysis involving 15 studies which assessed FAP expression in 11 solid cancers by IHC concluded that FAP positivity is found in 50–100% of patients, and a higher FAP expression is associated with (1) increased local tumor invasion, (2) increased risk of LN metastases, and (3) decreased survival, in particular in cases where FAP is expressed in the malignant cells [15]. FAP-specific inhibitors were developed and consecutively advanced into tumor-targeting radiopharmaceuticals leading to the recent introduction of [^68^ Ga]Ga-FAPI-04 PET/CT [10]. Initial results with [^68^ Ga]Ga-FAPI-04 PET/CT in patients suffering from overall 28 different kinds of cancer demonstrated high tracer uptake in sarcoma, cholangiocarcinoma, esophageal, breast, and lung cancer and intermediate uptake in hepatocellular, colorectal, head-neck, ovarian, pancreatic, and PC, providing the foundation to further explore FAP as a theranostic target in PC [10]. However, the role and expression of FAP in PC has not been comprehensively explored yet. Studying FAP expression in different stages of PC using IHC staining of established TMAs [11, 12], our results demonstrate that FAP expression increases with progression of disease. Corroborating our results that FAP is highly expressed in CRPC, we present three clinical case examples of patients with advanced PC undergoing [^68^ Ga]Ga-FAPI-04 PET/CT demonstrating high tracer uptake in the metastatic lesions. Interestingly, compared to benign and primary PC tissue, we also observed a rise in FAP expression in tissue samples from patients with neoadjuvant androgen deprivation therapy before radical prostatectomy, suggesting that neoadjuvant androgen deprivation therapy impacts the TME leading to increased FAP expression in some patients. To the best of our knowledge, only one other study has evaluated FAP expression in advanced PC so far. Hintz et al. analyzed publicly available RNA-seq datasets and found a significant increase in FAP mRNA expression in metastatic disease compared to primary PC. Additionally, in a mCRPC TMA, medium to strong IHC staining in metastatic lesions was observed compared to normal prostate. Furthermore, FAP gene expression was similar across all metastatic subtypes regardless of androgen receptor status or neuroendocrine differentiation [16]. These findings are in line with our results and further strengthen FAP as a potential diagnostic or therapeutic target in CRPC.

Our study has certain limitations. Benign TMA cores have been taken from RP specimens harboring PC elsewhere in the organ. The sample size might be too small to reveal a potentially existing significant difference in IHC staining between the non-significant groups. However, the broad confidence interval in NHT and NEPC samples indicates that some of these tumors demonstrate only low FAP expression. Detailed treatment data in CRPC and NEPC patients are lacking. The imaging case examples support the use of [^68^ Ga]Ga-FAPI-04 PET/CT in CRPC, but large-scale clinical studies will be needed to confirm its utility.

## Conclusion

FAP tissue expression supports further investigation of FAPI-molecular theranostics in CRPC.

## Supplementary Information

Below is the link to the electronic supplementary material.

Supplementary file1 (DOCX 14 KB)
