# Supplementary material for: High fibroblast-activation-protein expression in castration-resistant prostate cancer supports the use of FAPI-molecular theranostics
Source: Eur J Nucl Med Mol Imaging. 2021 Jul 5;49(1):385–9. doi: 10.1007/s00259-021-05423-y (PMC8712308; doi:10.1007/s00259-021-05423-y)
Supplement: Supplementary file 1 — Supplementary file1 (DOCX 14 KB) [file 259_2021_5423_MOESM1_ESM.docx]

Supplementary information

PET/CT imaging and evaluation

Imaging was performed on a Biograph mCT Flow scanner (Siemens). After non–contrast-enhanced low-dose CT (130 keV, 30 mAs, CareDose; reconstructed with a soft-tissue kernel to a slice thickness of 5 mm), PET was acquired in 3-dimensional mode (matrix, 200 × 200) using FlowMotion (Siemens). The emission data were corrected for random, scatter, and decay. Reconstruction was performed with an ordered-subset expectation maximization algorithm with 2 iterations/21 subsets and was Gauss-filtered to a transaxial resolution of 5 mm in full width at half maximum; attenuation correction was performed using the nonenhanced low-dose CT data. The PET scan was started 1 h after tracer injection. A 500 mL volume of saline with 20 mg of furosemide was infused from 15 min before to 30 min after tracer application. Tumor tracer uptake was quantified by SUVmean and SUVmax at 1 h after injection. For calculation of the SUV, circular regions of interest were drawn around the tumor lesions with focally increased uptake in transaxial slices and automatically adapted to a 3-dimensional volume of interest with e.soft software (Siemens) at a 60% isocontour. The unspecific background in blood pool (aortic vessel content) and muscle was quantified with a circular 2 cm diameter sphere.
